# Brevetoxin Aptamer Selection and Biolayer Interferometry Biosensor Application

**DOI:** 10.3390/toxins16100411

**Published:** 2024-09-24

**Authors:** Bo Hu, Sheng-Qun Ouyang, Yu-Ping Zhu, Xiao-Ling Lu, Zhe Ning, Bing-Hua Jiao, Liang-Hua Wang, Hao-Bing Yu, Xiao-Yu Liu

**Affiliations:** 1 Naval Medical Center of PLA, Naval Medical University, Shanghai 200433, China; hb8601@163.com (B.H.); ningzhe95@163.com (Z.N.); 2 College of Basic Medical Sciences, Naval Medical University, Shanghai 200433, China; shengqunouyang@163.com (S.-Q.O.); zhuyuping72@hotmail.com (Y.-P.Z.); luxiaoling80@126.com (X.-L.L.); bhjiao@smmu.edu.cn (B.-H.J.); lhwang@smmu.edu.cn (L.-H.W.)

**Keywords:** brevetoxin, aptamer, biolayer interferometry, aptasensor

## Abstract

Brevetoxins (PbTxs) are very potent marine neurotoxins that can cause an illness clinically described as neurologic shellfish poisoning (NSP). These toxins are cyclic polyether in chemistry and have increased their geographical distribution in the past 2 decades. However, the ethical problems as well as technical difficulties associated with currently employed analysis methods for marine toxins have spurred the quest for suitable alternatives to be applied in a regulatory monitoring regime. In this work, we reported the first instance of concurrent aptamer selection of Brevetoxin-1 (PbTx-1) and Brevetoxin-2 (PbTx-2) and constructed a biolayer interferometry (BLI) biosensor utilizing PbTx-1 aptamer as a specific recognition element. Through an in vitro selection process, we have, for the first time, successfully selected DNA aptamers with high affinity and specificity to PbTx-1 and PbTx-2 from a vast pool of random sequences. Among the selected aptamers, aptamer A5 exhibited the strongest binding affinity to PbTx-1, with an equilibrium dissociation constant (K_D_) of 2.56 μM. Subsequently, we optimized aptamer A5 by truncation to obtain the core sequence (A5-S3). Further refinement was achieved through mutations based on the predictions of a QGRS mapper, resulting in aptamer A5-S3G, which showed a significant increase in the K_D_ value by approximately 100-fold. Utilizing aptamer A5-S3G, we fabricated a label-free, real-time optical BLI aptasensor for the detection of PbTx-1. This aptasensor displayed a broad detection range from 100 nM to 4000 nM PbTx-1, with a linear range between 100 nM and 2000 nM, and a limit of detection (LOD) as low as 4.5 nM. Importantly, the aptasensor showed no cross-reactivity to PbTx-2 or other marine toxins, indicating a high level of specificity for PbTx-1. Moreover, the aptasensor exhibited excellent reproducibility and stability when applied for the detection of PbTx-1 in spiked shellfish samples. We strongly believe that this innovative aptasensor offers a promising alternative to traditional immunological methods for the specific and reliable detection of PbTx-1.

## 1. Introduction

**Figure 1:**
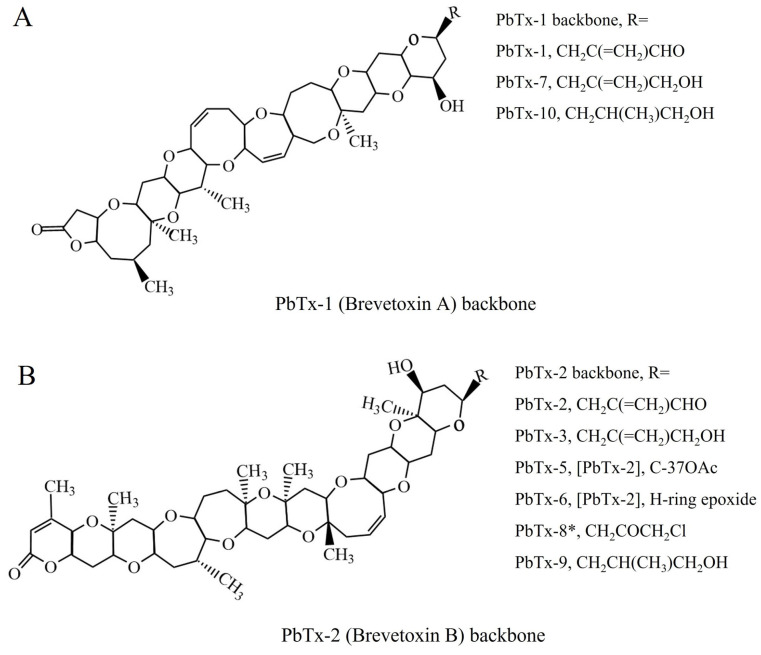
(**A**) Chemical structures of PbTx-1, PbTx-7, and PbTx-10. (**B**) Chemical structures of PbTx-2, PbTx-3, PbTx-5, PbTx-6, PbTx-8, and PbTx-9. *Denotes likely chemical artifact from extraction.

Brevetoxins (PbTxs), produced mainly by red tide dinoflagellates Karenia brevis, are typical marine toxins responsible for neurologic shellfish poisoning (NSP) [1,2]. From a chemical perspective, these toxins are potent cyclic polyether toxins with the characteristics of being tasteless, odorless, and resistant to heat and acidic conditions. The toxicity of PbTxs originates from their capacity to activate the voltage-gated sodium channels in nerve cells, which can result in nausea, diarrhea, headache, paralysis, coma, and so on [3]. The primary pathways for human exposure to PbTxs toxicity are the consumption of contaminated shellfish as well as the inhalation of aerosols in coastal areas [4]. In general, PbTxs have two distinct structural backbones, which are perceived to be the following two parent molecules: PbTx-1 and PbTx-2 (Figure 1) [3,5,6]. As is known, PbTx-1 is the most potent neurotoxin, while PbTx-2 is the most prevalent neurotoxin found in shellfish. Thus, it is of great importance to develop a rapid, sensitive, and specific method to detect PbTxs in contaminated seafood.

Mouse bioassay (MBA) is the most universally applied approach used to evaluate the toxicity of marine toxins, such as PbTxs [7]. Nevertheless, the specificity and sensitivity of this method is poor. Moreover, there are ethical concerns, hindering the development of this method. Analytical methods, like capillary electrophoresis [8], high-performance liquid chromatography (HPLC) [9], and liquid chromatography coupled to mass spectrometry (LC-MS) have been established [10]. However, these methods are usually time-consuming, costly, and inapplicable for on-site detection. Moreover, immunological methods, like enzyme-linked immunosorbent assay (ELISA) [11,12,13], have been developed for PbTxs detection after the successful production of antibodies specific to PbTxs. However, these techniques require highly trained personnel and expensive laboratory instruments. Meanwhile, it is impossible to prevent the risk of false positives and cross-reactivity. Taken together, these methods are unable to satisfy the demand for rapid and accurate on-site detection of PbTxs. Biosensors have the potential to overcome many of the disadvantages of other methods and are considered perfect substitutions for monitoring marine toxins [14]. The molecular recognition element is the core component of biosensors. As we know, antibodies are renowned recognition elements. However, they are always expensive, easily denatured, and sensitive to storage conditions. Therefore, it is urgent to find a new probe to establish detection methods that are economical, highly specific, and highly sensitive to PbTxs. 

In recent years, aptamers, novel molecular recognition elements, have attracted increasing attention in biosensor fabrication [15]. In nature, aptamers are single-stranded DNA (ssDNA) or RNA oligonucleotides obtained from an in vitro evolution-like process known as Systematic Evolution of Ligands by Exponential Enrichment (SELEX) [16]. They display almost all the merits of antibodies along with unique advantages. For example, they (1) can be synthesized in a simple, fast, and cost-effective manner using the well-established automated solid-phase synthesis method; (2) can bind specifically to a large variety of targets, including small molecules, proteins, and cells; (3) can be easily modified and immobilized on different surfaces using various functional tags; (4) are thermally stable when they are commercially shipped at ambient temperature. 

To date, plenty of aptasensors (biosensors using aptamers as molecular recognition elements) targeting marine toxins have been reported, such as saxitoxin [17,18], cylindrospermopsin [19], gonyautoxin 1/4 [20], palytoxin [21], and microcystin-LR [22,23]. In addition, Eissa et al. [24] and Tian et al. [25] have previously reported PbTx-2 aptamer selection, while several academics have developed PbTx-2 aptamer-based biosensors [26,27]. However, there have been no reports on aptamers specific to PbTx-1, the only natural toxin of the PbTxs, let alone aptasensor application.

In this work, we reported the first successful simultaneous selection of PbTx-1 and PbTx-2 aptamers and the application of a BLI aptasensor. Magnetic beads-based SELEX (MB-SELEX), a classical SELEX technique, was conducted to select aptamers specific to PbTx-1 and PbTx-2. After 18 rounds of selection, we obtained A5 (specific to PbTx-1) and B2 (specific to PbTx-2). Then, we truncated A5 and B2 based on their structures predicted on the mfold web server [28], and obtained aptamers A5-S1, A5-S2, A5-S3, B2-S1, and B2-S2. G-quadruple is a preferred secondary structure of aptamers and has been reported by many researchers [29,30,31]. Therefore, we further mutated A5-S3 and B2-S1 based on the results predicted by a QGRS mapper and obtained A5-S3G and B2-S1G, accordingly. The binding affinity between PbTx-1 and A5-S3G is about ten folds higher than that between PbTx-2 and A5-S1G. Given previous reports on PbTx-2 aptamer selection and biosensor application, we combined A5-S3G with biolayer interferometry (BLI), a highly sensitive, real-time, and label-free biosensor platform [32], to develop an aptasensor for PbTx-1 detection. This study hypothesizes that biotinylated aptamers of a proper concentration, when successfully immobilized on the surface of BLI biosensor, would induce a significant response when exposed to a positive sample.

## 2. Results

### 2.1. The Selection of DNA Aptamers Specific to PbTx-1 and PbTx-2

**Figure 2:**
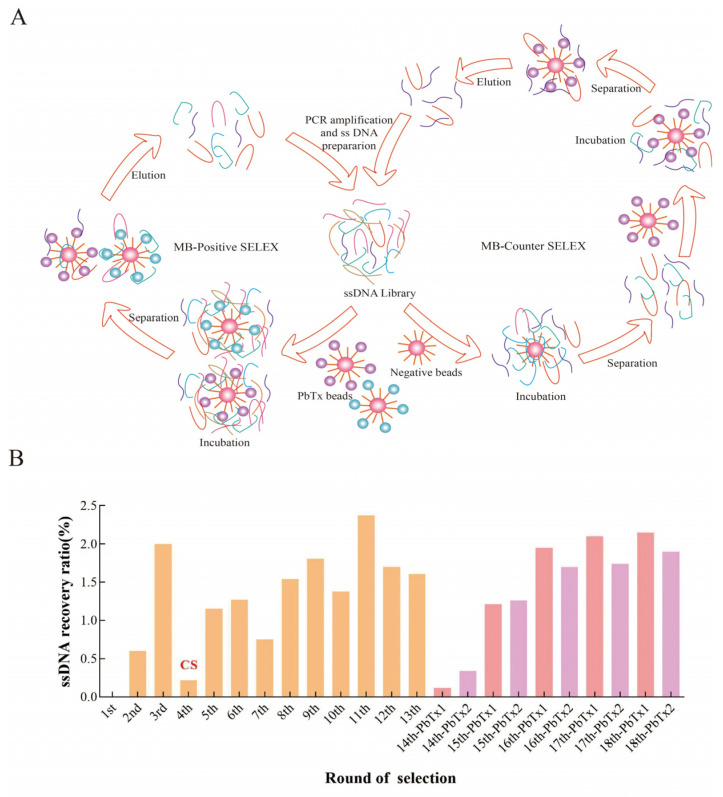
(**A**) Process of MB-SELEX. (**B**) Recovery ratio of ssDNA during MB-SELEX. The yellow columns represent the recovery ratio of ssDNA bound to PbTx-1 and PbTx-2. The pink columns represent the recovery ratio of ssDNA bound to PbTx-1. The purple columns represent the recovery ratio of ssDNA bound to PbTx-2. CS: Counter Selection.

We performed MB-SELEX, which comprised positive selection and counter selection illustrated in Figure 2A, according to the protocol depicted in Table S1. To improve the efficiency of the SELEX process, we added negative beads to remove ssDNA that non-specifically bound to the magnetic beads since round 4. After 13 rounds of selection, PbTx-1 and PbTx-2 beads were separately incubated with ssDNA to select aptamers specific to PbTx-1 and PbTx-2. The ssDNA recovery ratio showed no more obvious increases (Figure 2B). Therefore, we did not perform another round of selection. Furthermore, the enriched ssDNA was cloned and sequenced by Sangon Biotech Co., Ltd. (Shanghai, China). In total, we identified 30 sequences for PbTx-1 and PbTx-2, separately. We performed multiple sequence alignment using Clustal X 2.1 software. Sequences specific to PbTx-1 were grouped into three families (FA1-FA3, Figure S2), while those for PbTx-2 were classified into two families (FB1-FB2, Figure S2). We found some cross-homologous sequences among those families, and selected A5, B2, and B17 as the most representative sequences from each family.

### 2.2. Binding Affinity between Potential Aptamer and PbTx

**Figure 3:**
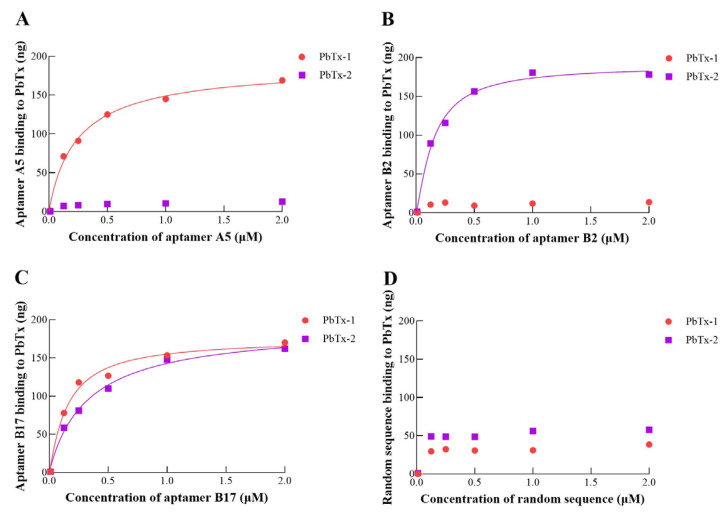
(**A**) Binding saturation curve of potential aptamer A5 to PbTx. (**B**) Binding saturation curve of potential aptamer B2 to PbTx. (**C**) Binding saturation curve of potential aptamer B17 to PbTx. (**D**) Binding saturation curve of the random sequence to PbTx.

A saturation curve was constructed to determine the binding affinity between candidate aptamer (A5, B2, and B17, along with a random sequence serving as a control) and PbTx (Figure 3). The results displayed that A5 had a high affinity for PbTx-1 (dissociation constant, i.e., K_D_ = 213 nM), while B2 exhibited a strong affinity for PbTx-2 (K_D_ = 114 nM). As anticipated, the random sequence displayed no interaction with either PbTx-1 or PbTx-2. However, B17 showed binding affinity for both PbTx-1 and PbTx-2, with K_D_ values of 168 nM and 250 nM, respectively. We hypothesized that (1) B17 bound to the common site of PbTx-1 and PbTx-2; and (2) B17 was a non-specific aptamer. As a result, B17 was excluded from further consideration as a potential aptamer, and our subsequent studies focused on aptamers A5 and B2.

### 2.3. Optimization of Aptamers

**Figure 4:**
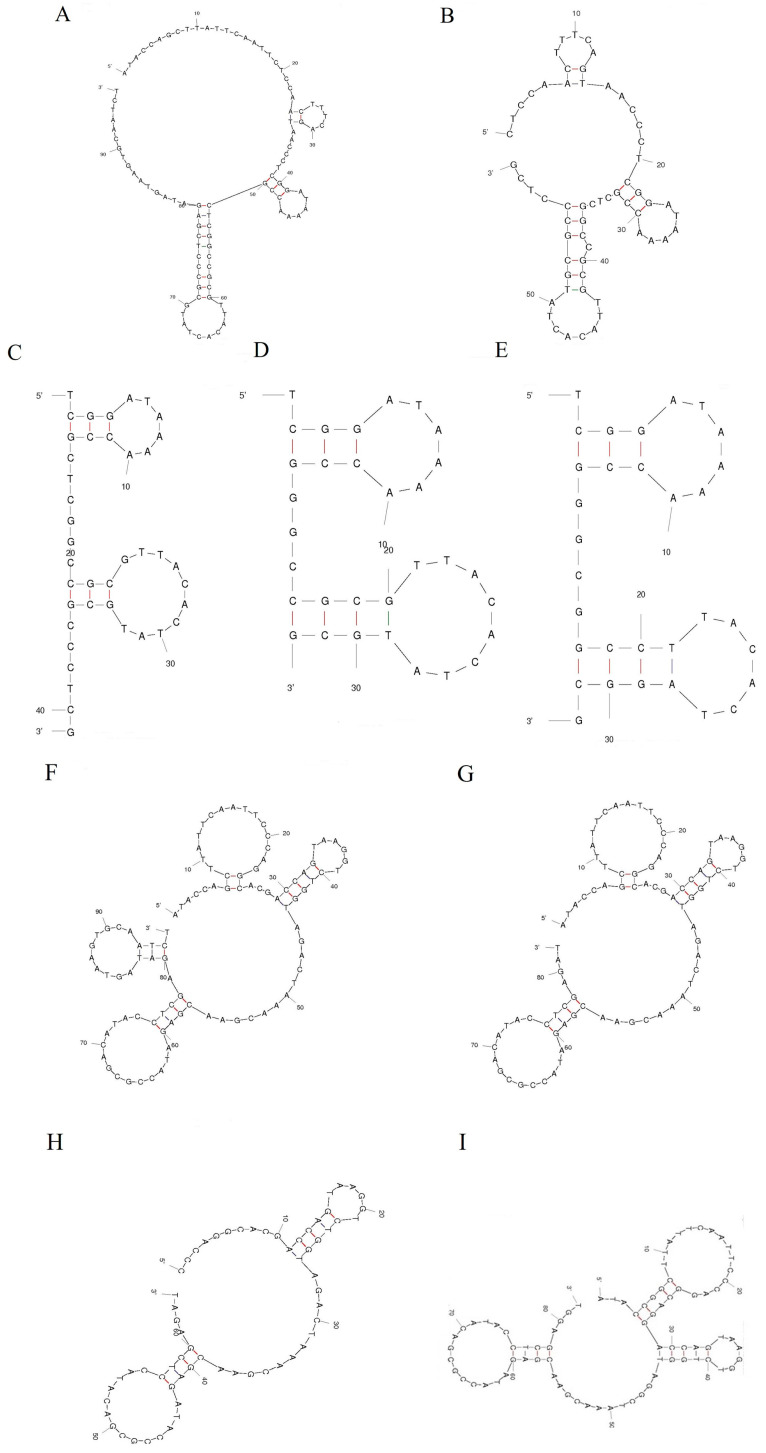
(**A**) Secondary structure of aptamer A5. (**B**) Secondary structure of aptamer A5-S1. (**C**) Secondary structure of aptamer A5-S2. (**D**) Secondary structure of aptamer A5-S3. (**E**) Secondary structure of aptamer A5-S3G. (**F**) Secondary structure of aptamer B2. (**G**) Secondary structure of aptamer B2-S1. (**H**) Secondary structure of aptamer B2-S2. (**I**) Secondary structure of aptamer B2-S1G.

We further truncated and mutated aptamers A5 and B2, which displayed competitive affinity and specificity for PbTx-1 and PbTx-2, separately. The truncated and mutated sequences of aptamer A5 and B2 are detailed in Table 1. We then predicted their secondary structures using the mfold web server (Figure 4). For aptamer A5, after primers at two ends (A5-S1) or its first stem ring (A5-S2 and A5-S3) was truncated, aptamers not only retained binding capability to PbTx-1 but also exhibited good affinity. G-quadruple is a favored secondary structure for aptamers and has been reported by many researchers. Consequently, we further mutated A5-S3 based on the prediction of the QGRS mapper and obtained A5-S3G. Expectedly, A5-S3G showed an enhanced binding affinity, with the K_D_ value increased from 2.56 μM to 72 nM. Regarding aptamer B2, there was no difference on its binding ability to PbTx-2 after deleting primers at the 3′ end (B2-S1). However, when the first ring at the 5′ end was truncated (B2-S2), the binding ability disappeared. Furthermore, we mutated B2-S1 to obtain a G-quadruple (B2-S1G). The results suggested that there was no significant change in the binding affinity.

### 2.4. Characterization of Aptamer A5-S3G

**Figure 5:**
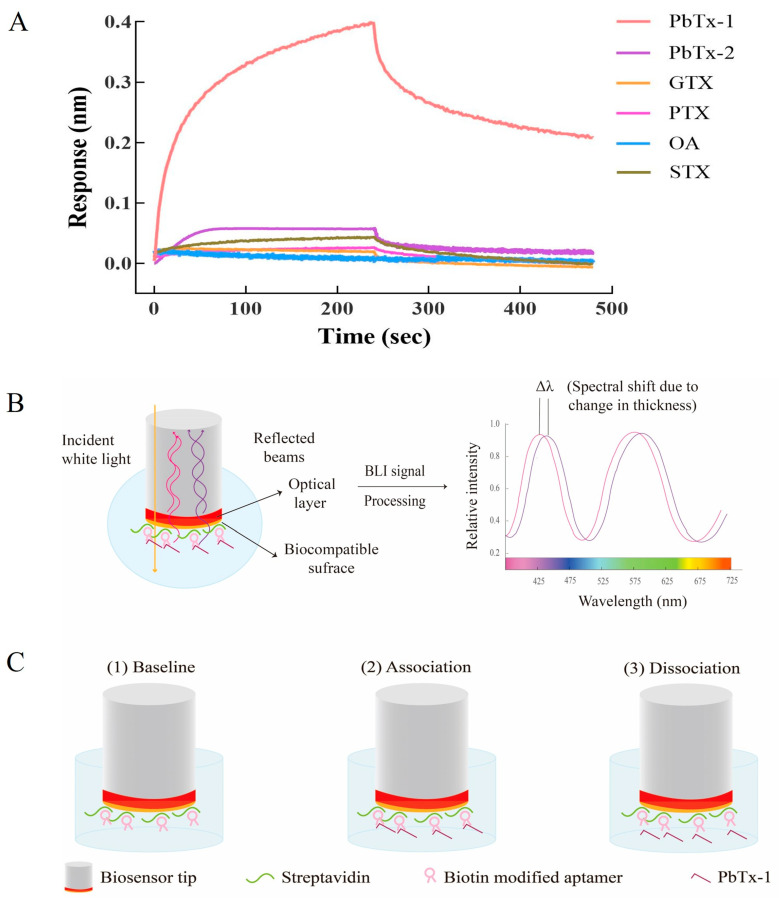
(**A**) Characterization of aptamer A5-S3G. (**B**) Principle of BLI aptasensor. (**C**) Working procedure of BLI aptasensor.

We further characterized the affinity and specificity of aptamer A5-S3G for PbTx-1 via BLI technology using an OctetRED 96 system (ForteBio, Shanghai, China) [33]. As shown in Table 1, the K_D_ value between aptamer A5-S3G and PbTx-1 was 72.3 nM. Moreover, it did not bind to other cyclic polyether toxins, like PbTx-2, gonyautoxin (GTX), palytoxin (PTX), okadaic acid (OA), and saxitoxin (STX) (Figure 5A). These results indicated that aptamer A5-S3G specifically and strongly binds to PbTx-1.

### 2.5. BLI Aptasensor for PbTx-1 Detection

Aptamer A5-S3G was further applied to fabricate a real-time and label-free optical BLI based aptasensor for PbTx-1 detection. BLI is a cutting-edge biosensor platform that uses biolayer interferometry technology to record the changes in the interfacial properties at the sensor surface during biological binding events, instead of handling samples without microfluidics. As shown in Figure 5B,C, a shift can be monitored in the interference spectrum and recorded as a change in wavelength over time when aptamers were immobilized on the surface of the biosensors. This wavelength shift (∆λ, Figure 5B) is applied to directly measure the change in optical thickness and mass density of the sensor layer. The interference spectrum and generated response curves were changed when PbTx-1, the target molecule, binds to or dissociates from the biosensor surface. The interference, which was a characteristic spectral signature, was captured and recorded in relative intensity units. Super streptavidin-coated (SSA) biosensors are widely applied in analyzing small molecules. Also, in this study, a larger number of aptamers can be immobilized on the spatial structure of the SSA sensor chip. Therefore, SSA biosensors were chosen for the online immobilization of aptamer A5-S3G.

**Figure 6:**
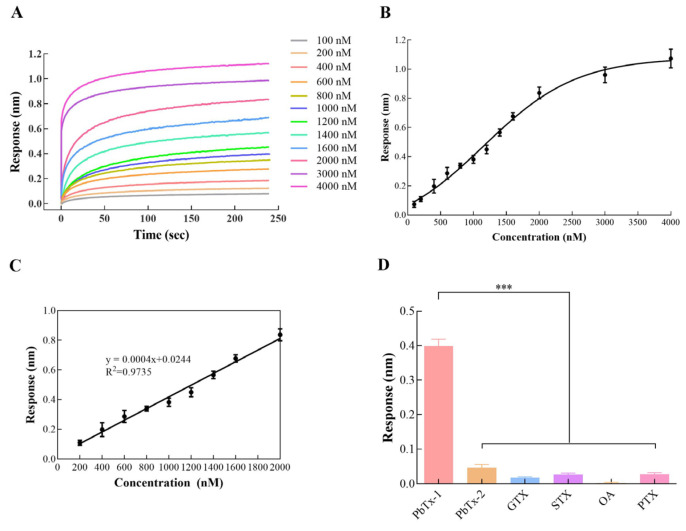
(**A**) The BLI aptasensor’s response to PbTx-1 at increased concentrations (100–4000 nM). (**B**) The calibration curve of the BLI aptasensor’s response to PbTx-1 at different concentrations (100–4000 nM). The error bars display standard deviations. (**C**) The linear range of the calibration curve for PbTx-1. The error bars display standard deviations. (**D**) The specificity of the aptasensor. The error bars display standard deviations. *** *p* < 0.001, vs. PbTx-1 (control).

In order to apply our BLI aptasensor to detect PbTx-1, we assessed the properties of our biosensor. Its real-time responses to increased concentrations of PbTx-1, from 100 nM to 4000 nM, were recorded. The changes in density and thickness of the biolayer surface were larger when the PbTx-1 concentration was increased. As a result, the BLI response was raised (Figure 6A). We drew a calibration curve of the BLI responses at 240 s against the PbTx-1 concentration, with each sample measured in triplicate (Figure 6B), and then fitted to a sigmoidal logistic five-parameter equation: y = (Rmax − Rmin)/[(1 + (x/EC50)^b^)]^n^ + Rmin. Here, Rmax and Rmin represented the maximum and minimum response, separately. EC50 (i.e., the median effect concentration) was the PbTx-1 concentration inducing 50% of the maximum response. b was the slope of the curve, while n was the correction factor. Based on the experimental data, we acquired the following equation: y = (0.836 − 0.251)/[(1 + (x/151.398)^−4.967^)]^0.231^ + 0.0251. Here, the correlation coefficient (R^2^) reached 0.999. Furthermore, the biosensor exhibited a good linear detection range between 100 nM and 2000 nM of PbTx-1, which could be represented by the linear regression equation y = 0.0004x + 0.0244 (R^2^ = 0.9735, Figure 6C). The limit of detection (LOD) was 4.5 nM (S/N = 3), where the noise level was the standard deviation of multiple measurements on blank samples (n = 10). The US Food and Drug Administration (FDA) and the US Environmental Protection Agency (EPA) have set a threshold concentration for PbTxs in food. According to these standards, the LOD is 0.8 ppm (0.8 mg/kg = 922.6 nM) [34]. It is significantly higher than that obtained by our aptasensor. The repeatability of our aptasensor was also measured by recording the signals after the addition of 1000 nM PbTx-1 on six separate occasions. The coefficient of variation (CV) was 2.3%, which suggested a good reproducibility of the aptasensor.

### 2.6. Detection of PbTx-1 in Shellfish Samples

In order to demonstrate the feasibility of PbTx-1 in complex matrix samples, spiked shellfish extracts were analyzed using the developed aptasensor. As shown in Table 2, a satisfactory recovery percentage was obtained for PbTx-1 in the shellfish extracts, indicating non-significant interference from the shellfish matrix on the aptasensor’s response. Thus, we can conclude that the aptasensor possesses promising features for practical use in the analysis of real-world samples.

## 3. Discussion

In total, we identified 30 sequences for PbTx-1 and PbTx-2, separately. Multiple-sequence alignment was performed with Clustal X 2.1 software. Sequences specific to PbTx-1 were classified into three families (FA1-FA3, Figure S2), while those specific to PbTx-2 were classified into two families (FB1-FB2, Figure S2). We found some cross-homologous sequences among those families, and selected A5, B2, and B17 as the most representative sequence from each family.

As expected, a random sequence displayed no interaction with either PbTx-1 or PbTx-2. However, B17 showed binding affinity to both PbTx-1 and PbTx-2, with K_D_ values of 168 nM and 250 nM, respectively. We hypothesized that (1) B17 bound to the common site of PbTx-1 and PbTx-2; and (2) B17 was a non-specific aptamer. As a result, we excluded it as a potential aptamer and focused on aptamers A5 and B2 in the following study.

In practical applications, specificity is a key property of an aptasensor, particularly in the presence of interference. Cross-reactivity experiments were conducted using 1000 nM of other cyclic polyether toxins, such as PbTx-2, GTX, PTX, OA, and STX. As displayed in Figure 6D, PbTx-1 caused a response of 0.398 nm. It was obviously higher than other toxin samples, which induced responses lower than 0.046 nm. These results suggested that PbTx-1 could be detected with good sensitivity. In other words, the aptasensor is highly specific.

Additionally, we summarized previous reports on antibodies or aptamer-based PbTxs detection. As shown in Table 3, the linear range of our aptasensor is not as extensive as those reported previously. Nevertheless, its LOD is not lower than those of all reported methods. More importantly, our aptasensor exhibits a superior LOD when compared to the thresholds defined by the FDA and EPA. More importantly, we assessed the capability of our aptasensor to detect PbTx-1 in complex matrix samples. The results suggested that our aptasensor has promising characteristics for practical use in the analysis of real-world samples.

## 4. Conclusions

In summary, we not only performed the selection of aptamers for PbTx-1 and PbTx-2 at the same time, but also developed the first aptasensor for PbTx-1 detection. The aptasensor exhibited commendable linearity within the range of 100 nM to 2000 nM of PbTx-1, coupled with a LOD as low as 4.5 nM. Additionally, the aptasensor showed high specificity and sensitivity. Consequently, we could conclude that the aptasensor may be used as an alternative to the traditional immunological methods, realizing the rapid and sensitive detection of PbTx-1. However, the aptasensor’s LOD is still higher than those of most aptasensor developed for PbTx-2 or PbTx-3 detection. Also, the complicated structure of the PbTx-1-A5-S3G complex and the binding mechanism of PbTx-1 to aptamer A5-S3G need to be further declared.

## 5. Materials and Methods

Materials and reagents applied throughout this study are described in detail in the Supplementary Materials.

### 5.1. Preparation of Magnetic Beads Coupled with PbTx

The preparation of PbTx-modified magnetic beads is shown in Figure S1. Details are described as below. A total of 175 μL buffer, which was composed of 20 mg N-succinimidyl-S-acetylthioacetate (SATA), 20 μL N, N-Dimethylformamide (DMF), and 180 μL phosphate-buffered saline (PBS) buffer, was added into amine beads dissolved in 1575 μL PBS buffer, and incubated with slow tilt rotation at room temperature for 0.5 h. After incubation, the amine beads were washed several times and resuspended in coupling buffer (350 μL coupling buffer for suspension). Then, 300 uL amine beads were put in 1500 μL hydroxylamine solution (0.1 mM, pH 7.2), and rotated slowly for 3 h at room temperature. The remaining 50 uL amine beads were further washed with PBS solution and resuspended in 50 μL binding buffer. They were stored at 4 °C and used for counter selection. The 600 μL PbTxs solution (125 ug/mL), which were dissolved in methanol, was incubated with 300 μL amine beads modified with mercapto groups like before. The mixture was rotated slowly for 3 h at room temperature. The efficiency of coupling was measured using the subtraction method using ELISA (Thermo Fisher Scientific, Shanghai, China). Next, N-Ethylmaleimide (NEM, 0.01 M, pH 7.0) was used to block unreacted mercapto groups existing on the surface of the amine beads. After another 2 h of end-over-end rotation at room temperature, the beads were extensively washed with binding buffer. Finally, PbTxs beads were stored in 300 μL binding buffer at 4 °C for positive selection.

### 5.2. In Vitro Selection of DNA Aptamers

The selection was performed according to the process described in Figure 2A, and details are depicted in the Supplementary Materials.

### 5.3. Saturation Curve of Potential PbTxs Aptamer 

The primary measurement of binding affinity between the potential aptamer and PbTxs was performed by drawing the saturation curve. In this regard, 5 μL PbTxs beads were incubated with their potential aptamer at different concentrations (0.01 μM, 0.125 μM, 0.25 μM, 0.5 μM, 1.0 μM, 2.0 μM), and the amount of aptamer binding to PbTxs beads was quantified using a Qubit^®^ 2.0 Fluorometer (Thermo Fisher Scientific, Shanghai, China). The K_D_ value was obtained by calculating the concentration of potential aptamer corresponding to its half-binding ability to PbTxs.

### 5.4. Determination of Aptamer Binding Affinity 

The binding affinity of the aptamers was further determined by BLI using an OctetRED 96 system (ForteBio, Shanghai, China) [33]. The principle and analysis procedures used herein were as detailed by Concepcion et al. [32]. As shown in Figure S3, the assay procedure includes five steps: (A) baseline (2 min); (B) loading (3 min); (C) washing (2 min); (D) association (2.5 min); (E) dissociation (2.5 min). The baseline solution (A, 200 μL, pH 7.5, 50 mM Tris-HCl, 150 mM NaCl, 2 mM MgCl_2_), loading solution (B, i.e., binding buffer), washing solution (C, i.e., binding buffer), association solution (D, i.e., binding buffer), and dissociation solution (E, i.e., binding buffer) were separately supplied to the corresponding wells in a 96-well microtiter plate (Figure S3). The response recorded on the reaction surface was normalized by subtracting the signal simultaneously acquired from the control surface. In this way, non-specific binding and buffer-induced interferometry spectrum shift were eliminated. Here, Octet Data Analysis Software CFR Part 11 Version 6.x was applied. The affinity parameter K_D_ was then calculated. A 1:1 binding mode with mass transfer fitting was employed to acquire the kinetic data.

### 5.5. BLI Aptasensor Preparation

SSA biosensors, which are widely applied for the analysis of small molecules, were employed for the online immobilization of the PbTx-1 aptamer -onto our aptasensors. The online preparation of the SSA aptasensors included three steps: (1) sensor activation (2 min); (2) biotinylated aptamer immobilization (5 min); and (3) washing (3 min). The activation solution (binding buffer, pH 7.5, 50 mM Tris-HCl, 150 mM NaCl, 2 mM MgCl2), immobilization solution (i.e., binding buffer), and washing solution (i.e., binding buffer) were utilized in their corresponding step. The responses of our aptasensor acquired from the reaction surface were adjusted by subtracting the signal data obtained from the control surface to remove non-specific binding and any buffer-induced interferometry spectrum shift.

### 5.6. PbTx-1 Extraction from Shellfish Tissue

Shellfish extracts were prepared following the protocol reported previously [35]. Briefly, the mussel samples were blended and extracted with methanol–water (80:20; 0.6 g/mL) for 5 min at 1800 rpm. Crude extracts were centrifuged for 10 min at 4000 rpm. One milliliter of extract was evaporated in a speed-vac concentrator, and the residue was resuspended in 1 mL of binding buffer at pH 7.4. The reconstituted solutions were passed through 0.45 µm pore nylon membrane filters and diluted 1:10 in binding buffer.

## Figures and Tables

**Table 1:** Dissociation constants (K_D_) between PbTx and optimized aptamer.

Aptamer	Target	Aptamer Sequence	K_D_ (μM)
A5	PbTx-1	ATACCAGCTTATTCAATTCTCCAACTTTCAGTAACCCTCGGATAAAACCGCTCGGCCGCGTTACACTATGCGCCCTCGAGATAGTAAGTGCAATCT	2.56
A5-S1	PbTx-1	CTCCAACTTTCAGTAACCCTCGGATAAAACCGCTCGGCCGCGTTACACTATGCGCCCTCG	1.66
A5-S2	PbTx-1	TCGGATAAAACCGCTCGGCCGCGTTACACTATGCGCCCTCG	0.343
A5-S3	PbTx-1	TCGGATAAAACCGGGCCGCGTTACACTATGCG	0.178
A5-S3G	PbTx-1	TCGGATAAAACCGGGCGGCGTTACACTAGGCG	0.072
B2	PbTx-2	ATACCAGCTTATTCAATTCCCAGGCACGACCAGTAAGGTCTGGTAGACTAAACGAACGAGATACCGCGACATACCTCGAGATAGTAAGTGCAATCT	2.21
B2-S1	PbTx-2	ATACCAGCTTATTCAATTCCCAGGCACGACCAGTAAGGTCTGGTAGACTAAACGAACGAGATACCGCGACATACCTCGAGAT	1.63
B2-S2	PbTx-2	CCCAGGCACGACCAGTAAGGTCTGGTAGACTAAACGAACGAGATACCGCGACATACCTCGAGAT	---
B2-S1G	PbTx-2	ATACCGGCTTATTCAATTCCCAGGCAGGACCAGTAAGGTCTGGTAGGCTAAACGAACGAGATACCGCGACATACCTCGAGGT	0.76

**Table 2:** Recovery studies of shellfish samples at three levels using the aptasensor (n = 3).

Shellfish Samples	Spiked Shellfish PbTx-1 (ng/mL)	Recovery (%)	CV (%)
1	200	104.09	1.62
2	600	106.87	1.83
3	1400	108.32	3.72

**Table 3:** Summary of antibody or aptamer-based PbTxs detection.

Analytical Techniques ^a^	Targets	Linear Range	LOD	Reference
ELISA	PbTx-2	3.51–225 nM	Not reported	[25]
ELISA	PbTx-1	15.70–295 nM	15.70 nM	[11]
ELISA	PbTx-3	0.05–2.25 nM	Not reported	[13]
EIS	PbTx-2/PbTx-3	Not reported	0.12 nM	[24]
QCM	PbTx-2	10–1000 nM	220 nM	[27]
SE	PbTx-2	0.05–1600 nM	1.48 nM	[26]
TIRE	PbTx-2	0.50–2000 nM	0.80 nM	[26]
BLI	PbTx-1	100–2000 nM	4.50 nM	This work

^a^ ELISA—enzyme-linked immunosorbent assay; QCM—quartz crystal resonator; SE—spectroscopic ellipsometry; EIS—electrochemical impedance spectroscopy; TIRE—attenuated internal reflection spectroscopic ellipsometry; BLI—biolayer interferometry.

## Data Availability

The raw data supporting the conclusions of this article will be made available by the authors on request.

## Supplementary Materials

The following supporting information can be downloaded at https://www.mdpi.com/article/10.3390/toxins16100411/s1: Figure S1: Preparation of PbTx-modified magnetic beads; Figure S2: Multiple-sequence alignment of the selected sequences by MB-SELEX; Figure S3: Principal process of BLI assay; Table S1: Summary of selection protocol for MB-SELEX.
