# Supplementary material for: Brevetoxin Aptamer Selection and Biolayer Interferometry Biosensor Application
Source: Toxins (Basel). 2024 Sep 24;16(10):411. doi: 10.3390/toxins16100411 (PMC11510897; doi:10.3390/toxins16100411)
Supplement: Supplementary file 1 [file toxins-16-00411-s001.zip › toxins-3201511-Supplementary Materials.pdf]

Supplementary Materials

Brevetoxin Aptamer Selection and Biolayer Interferometry Biosensor Application

Bo Hu [1,†], Sheng-Qun Ouyang [2,†], Yu-Ping Zhu [2,†], Xiao-Ling Lu [2], Zhe Ning [1], Bing-Hua Jiao [2], Liang-Hua Wang [2], Hao-Bing Yu [1,*] and Xiao-Yu Liu [1,*]

[1] Naval Medical Center of PLA, Naval Medical University, Shanghai 200433, China; hb8601@163.com (B.H.); ningzhe95@163.com (Z.N.)

[2] College of Basic Medical Sciences, Naval Medical University, Shanghai 200433, China; shengqunouyang@163.com (S.-Q.O.); zhuyuping72@hotmail.com (Y.-P.Z.); luxiaoling80@126.com (X.-L.L.); bhjiao@smmu.edu.cn (B.-H.J.); lhwang@smmu.edu.cn (L.-H.W.)

* Correspondence: yuhaobing1986@126.com (H.-B.Y.); biolxy@163.com (X.-Y.L.); Tel.: +86-021-81883270 (H.-B.Y.); +86-13361967536 (X.-Y.L.)

† These authors contributed equally to this work.

Materials and Methods

1. Materials and Reagents

All nucleic acid sequences were custom-synthesized by Sangon Biotech Co. Ltd (Shanghai, China). Brevetoxin A (PbTx-1), Brevetoxin B (PbTx-2), Gonyautoxin (GTX), palytoxin (PTX), okadaic acid (OA) and saxitoxin (STX) were obtained from Taiwan Algal Science, Inc. (Taiwan). Dynabeads® M-270 Amine and the Qubit® ss DNA Assay Kit were purchased from Invitrogen. N-succinimidyl-S-acetylthioacetate (SATA), N, N-Dimethylformamide (DMF), N-Ethylmaleimide (NEM) and other chemical agents were purchased from Sigma-Aldrich Co. LLC (mainland, China). Dr. Gen TLE® Precipitation Carrier and 20 bp DNA Ladder (Dye Plus) were procured from TaKaRa Bio, Inc. (Dalian, China). GoTaqHot® Start Colorless Master Mix was purchased from Promega Corporation (Shanghai, China). Uera (ultra-pure grade) was obtained from AMRESCO (Beijing, China). Bio Gel P-2 was obtained from Bio-Rad (Hercules, USA) and 3-mercaptopropionic acid from Fluka (Germany). BLI sensor chips were obtained from Forte Bio (Shanghai, China). DNA-PAGE buffer, phosphate buffer (PBS), and binding buffer (pH 7.5, 50 mM Tris-HCl, 150 mM NaCl, 2 mM $MgCl_2$) were procured from Tiandz (Beijing, China). Coupling buffer was PBS buffer supplied with 1% DMF and was used to couple unsaturated aldehydes and ketones in PbTx and amine beads decorated with mercapto groups. Binding buffer was used for the aptamer selection, biolayer interferometry (BLI) experiment. The elution buffer was binding buffer with 7 mM urea in it. PBS was used for the coupling between unsaturated aldehydes and ketones in PbTx-1 and the amine beads decorated with mercapto groups. All solutions were prepared using Milli-Q ultrapure water.

2. Random ssDNA Library and Primer Set

A random ssDNA library containing 5nmol ssDNA oligonucleotides (3.01×10^{15}) was chemically synthesized and purified by Sangon Biotech Co. Ltd (Shanghai, China). The ssDNA library was made up of a central region of 60 random nucleotides flanked by 18 fixed nucleotides at both 3’ and 5’ end. Library sequence: 5’-ATACCAGCTTATTCAATT-N60- AGATAGTAAGTGCAATCT-3’. Fixed nucleotides

are primer binding sites for amplification of the library sequence. Forward primer: 5'-ATACCAGCTTATTCAATT-3'; Reverse primer A: 5' -AGATTGCACTTACTATCT-3'; Reverse primer B: 5'-poly (dA20)-PEG6-AGATTGCACTTACTATCT-3'. Unmodified reverse primer was used for PCR amplification and cloning when SELEX rounds were performed. Modified reverse primer was designed for the separation of single stranded DNA from PCR amplified double stranded DNA.

3. In Vitro Selection of the DNA Aptamers

The positive and counter MB-SELEX processes are illustrated in Fig 2, following the protocol detailed in Table S1. A certain amount of ssDNA oligonucleotides (5 nmol in the first selection round; 200 pmol in the second and third selection round; 120 pmol in the left selection round.) were dissolved in binding buffer. Before selection, they were heated to 95 °C for 10 min and cooled in an ice bath for 5 min to form the optimal structural conformation. Simultaneously, the PbTx-1 and PbTx-2 beads were washed several times with binding buffer. The prepared library and 5 μL washed PbTx-1 and PbTx-2 beads were immediately added into a microcentrifuge tube. Binding buffer was further added to have a total volume of 300 μL. After incubating the mixture for 2 h with end-over-end rotation at room temperature, the beads were washed several times with 500 μL binding buffer until no DNA could be detected in the supernatant. The ssDNA bound to PbTx-1 and PbTx-2 beads were eluted with 500 μL elution buffer three times with shaking and heating at 95 °C for 20 min. The eluted ssDNA was recovered, quantified and amplified. For the counter MB-SELEX screening, the ssDNA library, subjected to the same heating and cooling treatment as before, was incubated with the negative beads. The ssDNA in the supernatant was then recovered and quantified. After another heating and cooling treatment, the ssDNA was incubated with PbTx-1 and PbTx-2 beads. After incubation at room temperature, the PbTx-1 and PbTx-2 beads were washed and eluted as before. The eluted ssDNA was quantified using a Qubit® 2.0 Fluorometer. Since round 14, PbTx-1 and PbTx-2 beads were separately incubated with ssDNA to select aptamers specific to PbTx-1 or PbTx-2.

Table S1. Summary of selection protocol for MB-SELEX

Selection round	Amount of ssDNA pool (nmol)	Incubation beads
1	5	PbTx-1 and PbTx-2 beads
2-3	0.2	PbTx-1 and PbTx-2 beads
4-13	0.12	PbTx-1, PbTx-2 and negative beads
14-18	0.12	PbTx-1 or PbTx-2 beads

4. Amplification and Purification of the ss DNA

The enriched pools were amplified by PCR in 40 parallel 50 μL reactions, each containing Go Taq® Hot Start DNA Polymerase and Colorless Go Taq® Reaction Buffer (pH 8.5), 200 μM dNTP, 2 mM $MgCl_2$ and 0.5 μM forward primer and reverse primer B. PCR conditions were as below: 94 °C for 3 min, followed by 25 cycles of 95 °C for 30 s, 60 °C for 30 s, 72 °C for 30 s, and a final extension step of 5 min at 72 °C. The enriched ssDNA existing in PCR products were separated by 12% urea denaturing PAGE, and recovered by boiling the gel band. Next, the collected ssDNA was purified by ethanol precipitation and dried at room temperature for 30 min. Finally, the dried ssDNA powders were dissolved in binding buffer and used for the next selection round.

5. Cloning and Sequencing of Selected DNA Aptamers

After 18 rounds of selection, ssDNA pool selected by MB-SELEX was amplified with forward primer and reverse primer A (unmodified reverse primer). PCR products were purified by ethanol precipitation. The collected double strand DNA (dsDNA) were cloned and sequenced by Sangon Biotech Co. Ltd (Shanghai, China). The target ssDNA sequences were aligned and analyzed by the Clustal X software. Their secondary structures were predicted by the mfold Web Server (http: //mfold. rna.albany. edu/? q=m fold).

Supporting Results

1. Preparation of PbTx-modified Magnetic Beads

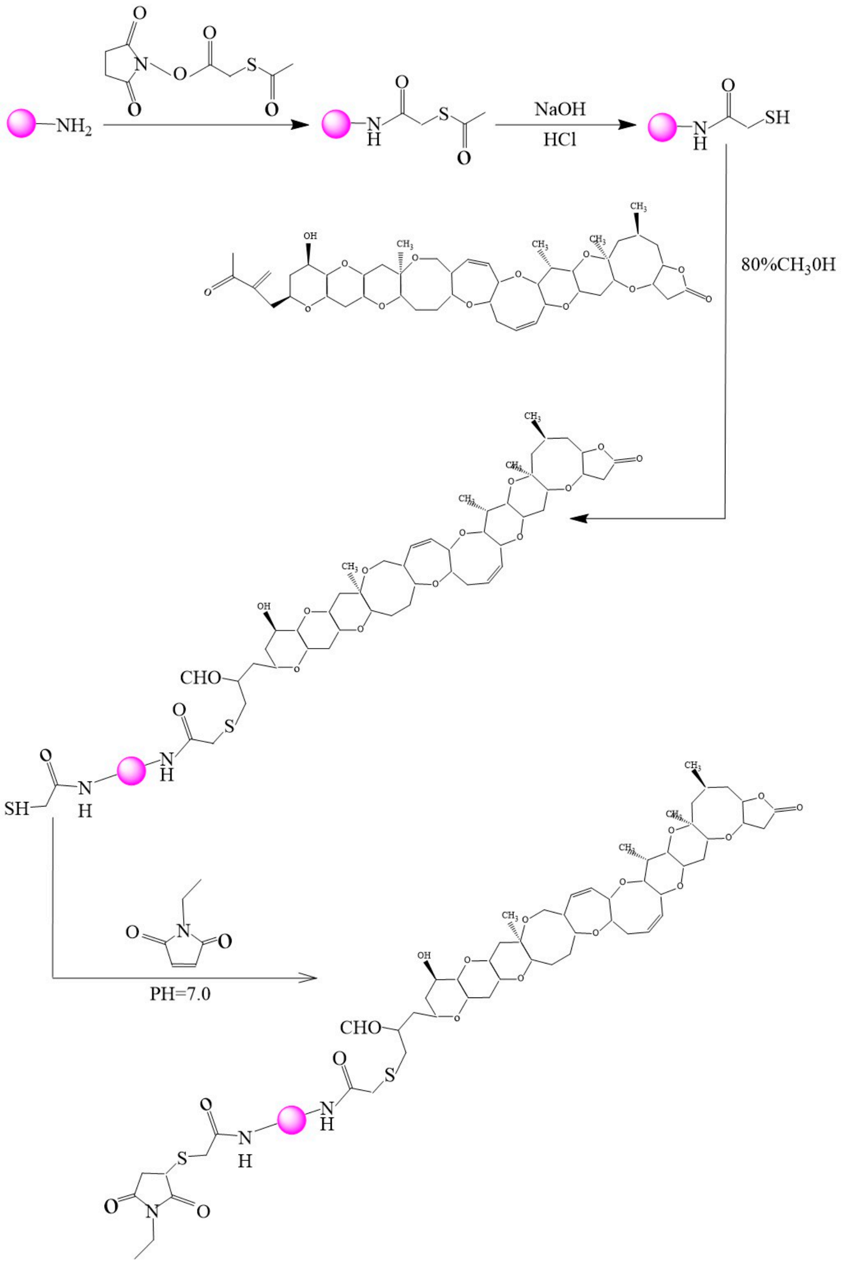

Figure S1. Preparation of PbTx-modified magnetic beads. Step 1-2: Modification of amine beads with mercapto groups. Step 3: Coupling between unsaturated aldehydes and ketones in PbTx and the amine beads decorated with mercapto groups. Step 4: Blocking of the unreacted mercapto groups existing on the surface of amine beads.

2. Multiple Sequence Alignment of The Selected Sequences by MB-SELEX

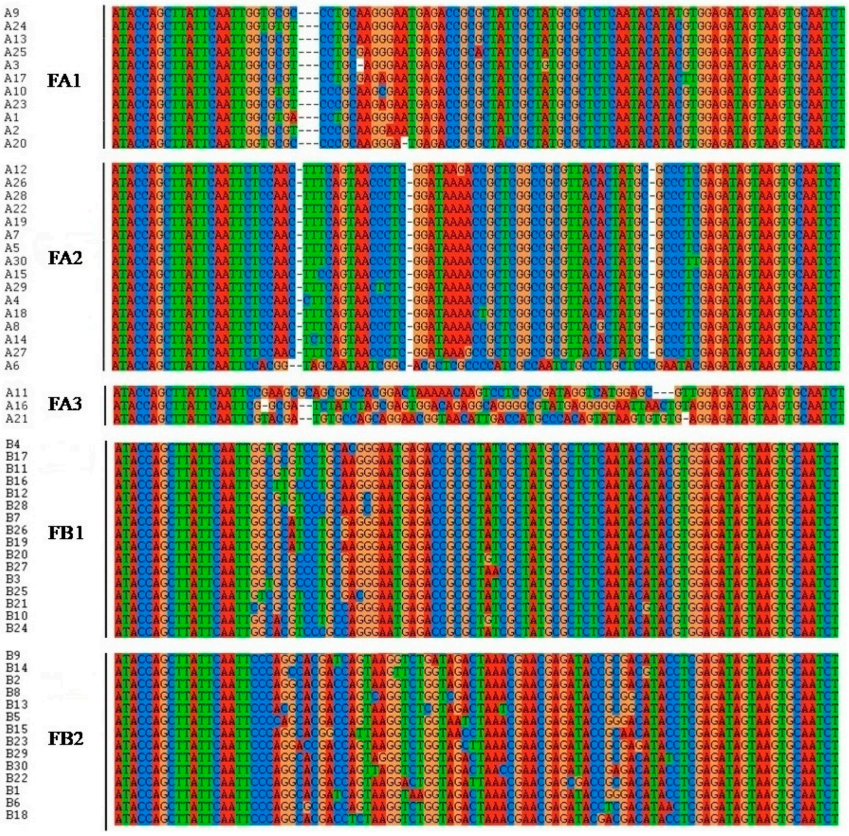

Figure S2. Multiple sequence alignment of sequences for PbTx-1 and PbTx-2. Sequences for PbTx-1 were grouped into family FA1, FA2, and FA3, while those for PbTx-2 were grouped into family FB1 and FB2.

3. Principal Process of BLI Assay

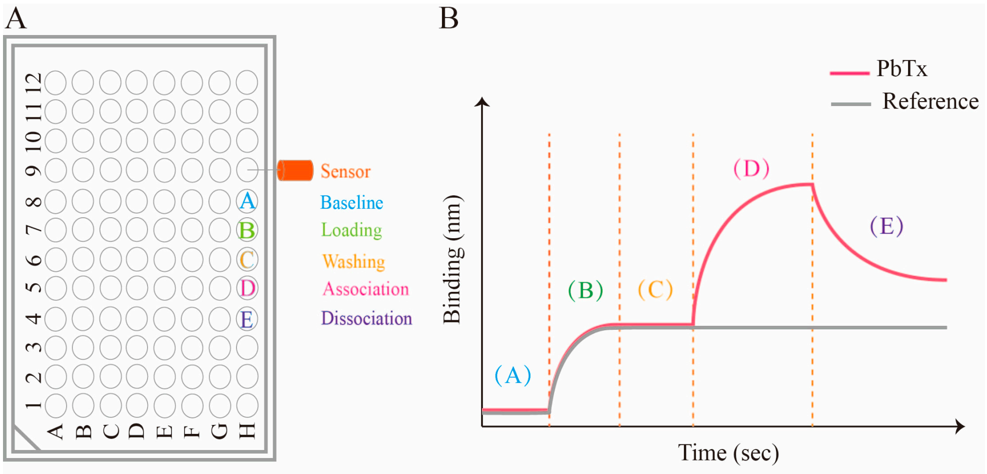

Figure S3. BLI assay included five steps: (A) baseline; (B) loading; (C) washing; (D) association; (E) baseline. A reference sensor was always required as a control in every assay.
